# New evidence calls into question NICE's endocarditis prevention guidance

**DOI:** 10.1038/s41415-024-7344-5

**Published:** 2024-05-10

**Authors:** Martin Thornhill, Bernard Prendergast, Mark Dayer, Ash Frisby, Peter Lockhart, Larry M. Baddour

**Affiliations:** 4141556414001 Professor of Translational Research in Dentistry, Unit of Oral and Maxillofacial Medicine, Surgery and Pathology, School of Clinical Dentistry, University of Sheffield, Sheffield, UK; 4141556414002 Professor of Cardiology, Guy´s and St Thomas´ Hospital, London and Chair of Cardiology, Cleveland Clinic, London, UK; 4141556414003 Professor and Consultant Cardiologist, Cardiovascular Research Institute, Mater Private Network, Dublin, Ireland; 4141556414004 Patient Advocate, London, UK; 4141556414005 Research Professor, Department of Oral Medicine/Oral and Maxillofacial Surgery, Atrium Health´s Carolinas Medical Centre, Charlotte, North Carolina, USA; Adjunct Professor, Department of Otolaryngology, Wake Forest University School of Medicine, North Carolina, USA; 4141556414006 Professor Emeritus, Division of Public Health, Infectious Diseases and Occupational Health, Departments of Medicine and Cardiovascular Medicine, Mayo Clinic College of Medicine and Science, Rochester, Minnesota, USA

## Abstract

In 2008, National Institute for Health and Care Excellence (NICE) guidelines recommended against the use of antibiotic prophylaxis (AP) before invasive dental procedures (IDPs) to prevent infective endocarditis (IE). They did so because of lack of AP efficacy evidence and adverse reaction concerns. Consequently, NICE concluded AP was not cost-effective and should not be recommended. In 2015, NICE reviewed its guidance and continued to recommend against AP. However, it subsequently changed its wording to ‘antibiotic prophylaxis against infective endocarditis is not routinely recommended'. The lack of explanation of what constituted routinely (and not routinely), or how to manage non-routine patients, caused enormous confusion and NICE remained out of step with all major international guideline committees who continued to recommend AP for those at high risk.

Since the 2015 guideline review, new data have confirmed an association between IDPs and subsequent IE and demonstrated AP efficacy in reducing IE risk following IDPs in high-risk patients. New evidence also shows that in high-risk patients, the IE risk following IDPs substantially exceeds any adverse reaction risk, and that AP is therefore highly cost-effective. Given the new evidence, a NICE guideline review would seem appropriate so that UK high-risk patients can receive the same protection afforded high-risk patients in the rest of the world.

## Key points

- National Institute for Health and Care Excellence (NICE) guidelines for the prevention of infective endocarditis (IE) are almost unique worldwide in not recommending routine antibiotic prophylaxis (AP) before invasive dental procedures (IDPs) in patients at high risk of IE.
- Recent data confirm an association between IDPs and subsequent IE and show that AP is safe, cost-effective and significantly reduces the incidence of IE following IDPs in those at high IE risk.
- Re-introduction of AP for those at high risk could potentially prevent 41-261 IE cases in the UK each year (including ~12-78 deaths) and produce substantial cost savings for the NHS.
- Given this new evidence, it is time for NICE to review its recommendations so that patients in the UK receive the same protection against IE as others in the rest of the world.

## Background

Over the past century, dentistry has transformed oral health in the UK and serious morbidity or death from oral disease or dental procedures is now extremely rare. However, infective endocarditis (IE), secondary to oral bacteria, remains a concern and it is important that dentists and their patients receive clear, unambiguous guidance about how to protect their patients from this life-threatening condition.

**Fig. 1:**
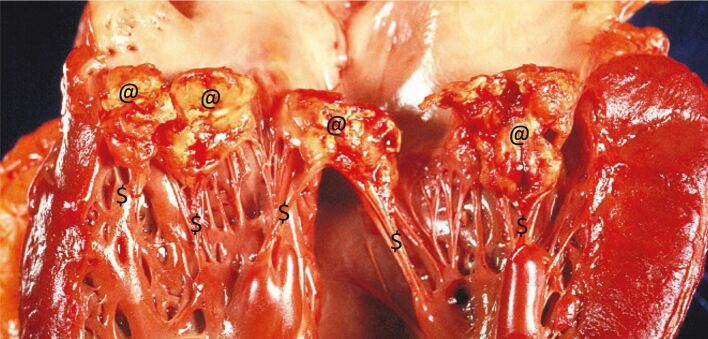
IE with mitral valve vegetations, scarred and perforated valve leaflets^@^ and inflamed and scarred chordae tendineae^$^

IE is caused by bacteria entering the circulation and producing a heart valve infection in susceptible individuals. This stimulates the growth of vegetations (infected scar tissue) on the heart valves that cause valvular obstruction, incompetence, or perforation, with resulting heart failure (Fig. 1). Fragments of these vegetations may also embolise and lodge in distant blood vessels to cause strokes, brain abscesses, renal damage and other peripheral complications.

Diagnosis is often difficult. Initial symptoms are non-specific (low-grade fever and malaise) and diagnosis is often delayed until the patient is severely ill and admitted urgently to hospital. Once IE is suspected, it may be confirmed by blood cultures and echocardiography, but 15-20% of IE patients still die during their initial hospital admission (and another 10-15% within the first year).^1^^,^^2^ Those who survive often have severe ongoing disabilities that blight their lives and require repeated medical interventions, and they remain at high risk for future episodes of IE (Box 1).

Worryingly, IE incidence is increasing in the UK^3^^,^^4^^,^^5^ and across Europe.^6^ Multiple factors are likely to be responsible, including: an ageing population; improved diagnostic techniques; the increasing number of patients with cardiac diseases treated with prosthetic heart valves and other cardiac devices that are associated with a high risk for IE; and reduction in the use of antibiotic prophylaxis (AP) before invasive dental procedures (IDPs). For a definition of what dental procedures are considered IDPs by most guideline committees, see Box 1 in our accompanying article: ‘Prevention of infective endocarditis in at risk patients: how should dentists proceed in 2024?'.^7^

A century ago, in 1923, Lewis and Grant first suggested that IE might result from the bacteraemia caused by IDPs.^8^ In 1935, Okell and Elliott noted that most patients had oral viridans group streptococci (OVGS) in their blood following dental extraction and linked this directly to the aetiology of IE.^9^ They also noted that bacteraemia was most likely to occur in those with poor oral hygiene. Even now, around 35-45% of IE cases are caused by bacteria found most commonly in the mouth (including OVGS).^3^

Box 1 Infective endocarditis - basic facts (for more details see Table 4 in the accompanying article)^7^

- During 2021-22 there were 12,707 hospital admissions in the UK where IE was the principal diagnosis (NHS Digital Hospital Admissions data)
- IE incidence is rising in the UK^3^^,^^4^^,^^5^
- 15-20% of IE patients die during their initial hospital admission^1^^,^^2^
- A further 10-15% die over the following year^1^^,^^2^
- 35-45% of cases are caused by OVGS^3^
- 40-45% require surgery during the initial hospital admission (often involving prosthetic replacement of one or more heart valves) and a further 10% need surgery in the ensuing year^1^^,^^2^
- Many survivors will have significantly reduced quality and length of life^1^^,^^2^
- Presentation can be subtle - malaise, weight loss and fever are the most common presenting symptoms.

## International guidelines and the UK perspective

In 1955, the American Heart Association (AHA) produced the first IE prevention guidelines, recommending that individuals at increased IE risk should receive AP before IDPs to reduce their risk of developing IE.^10^ Similar guidelines followed soon after in the UK, Europe and the rest of the world. By the early 2000s, international guideline committees were consistently recommending AP in all those at increased IE risk (that is, those at moderate and high risk [Table 1]). However, there were justifiable concerns about the lack of evidence for AP efficacy, the risk of adverse reactions to AP, and the possibility of antimicrobial resistance resulting from unnecessary antibiotic use. As a consequence, the British Society for Antimicrobial Chemotherapy (BSAC) broke rank in 2006 and were the first to propose that AP use should be restricted to those at highest risk for IE (Table 1), and, in other words, should cease for those at moderate risk^11^ (representing an approximate 90% reduction in the number of patients for whom AP was recommended).^12^ Unfortunately, this recommendation was met with strong reaction and was condemned as excessive by several national cardiological organisations,^13^^,^^14^ resulting in referral to the National Institute for Health and Care Excellence (NICE). Considerable surprise ensued in 2008 when NICE went much further than BSAC and recommended that use of AP to prevent IE should cease completely^15^ - a decision that conflicted with the recommendations from all other international guideline committees, including the AHA^16^ and European Society for Cardiology (ESC),^17^ who, like BSAC, recommended that AP cover should continue for IDPs in those at high risk of IE.

**Table 1:** Cardiac conditions that identify individuals at high, moderate or low risk of developing infective endocarditis (based on the AHA,^16^^,^^24^^,^^47^ BSAC^11^ and ESC^17^^,^^19^^,^^25^^,^^48^guideline definitions of those at high-, moderate- or low-risk of IE)

**High risk**
Previous history of IE
Presence of prosthetic cardiac valve (including transcatheter devices)
Prosthetic material used for valve repair (including annuloplasty and transcatheter devices)
Un-repaired cyanotic congenital heart disease
Congenital heart disease intervention using palliative shunts or conduits
Completely repaired congenital heart defect (using prosthetic material or surgical/transcatheter device)*
Ventricular assist devices**
**Moderate risk (also known as intermediate risk)**
Rheumatic heart disease
Non-rheumatic valve disease (including mitral valve prolapse)
Congenital valve anomalies (including bicuspid aortic valve)
Cardiac implantable electronic devices (CIED) for example, pacemaker or defibrillator**
Hypertrophic cardiomyopathy
**Low risk**
Patients with none of the above high- or moderate-risk conditions
^Key:^ * = Only for the first six months after the procedure ** = New moderate- and high-risk conditions featured in 2023 ESC guidance (but not in earlier SDCEP advice)^25^

In 2015, an observational study published in *The Lancet *reported an 88% fall in AP prescribing in England following the 2008 NICE guidance, accompanied by a significant increase in IE incidence.^3^ Prompted by these findings, NICE and ESC reviewed their guidance. NICE methodology at that time required the availability of new randomised controlled trial (RCT) evidence (which *The Lancet* study was not) to mandate changing recommendations. Accordingly, NICE reiterated its guidance that ‘AP against IE is not recommended for people undergoing dental procedures'.^18^ In contrast, the ESC reviewed exactly the same evidence and maintained that use of AP should continue in patients at high IE risk.^19^

## Change in wording of NICE guidance that resulted in confusion

Subsequently, in 2016, without any announcement, explanation or review, NICE changed the wording of its guidance, adding the word ‘routinely' and stating that ‘antibiotic prophylaxis against infective endocarditis is not routinely recommended for people undergoing dental procedures'.^15^

This change caused confusion for dentists, cardiologists and their patients. The change implied that AP was recommended in certain non-routine situations, but NICE provided no guidance as to which patients or dental procedures should be considered non-routine (or what AP regimen should be used). In other words, unlike their US and European counterparts, the NICE guidelines provided no clinically useful guidance for dentists, cardiologists, or their patients. In frustration, many cardiology centres (such as the Royal Brompton Hospital Adult Congenital Heart Unit),^20^ took matters into their own hands and adopted the ESC guidance instead. This divided stance, with some cardiologists following ESC guidance, while others adhered to NICE guidance, resulted in inevitable further confusion for dentists and their patients.

In a noble attempt to address this confusion, the Scottish Dental Clinical Effectiveness Programme (SDCEP) produced advice for dentists in 2018 on how to implement the NICE guidelines, advising that ‘the vast majority of patients at increased risk of IE will not be prescribed AP. However, for a very small number of patients, it may be prudent to consider AP (non-routine management) in consultation with the patient and their cardiologist or cardiac surgeon'.^21^ Although the SDCEP categorisation of patients for whom AP should be considered was the same as that recommended by the ESC and AHA (Table 1), they advised dentists to only consider AP if endorsed by the patient's cardiologist or cardiac surgeon. In this instance, SDCEP advised dentists to ‘discuss the potential benefits and risks of prophylaxis for IDPs with the patient to allow them to make an informed decision about whether prophylaxis is right for them'. Unfortunately, the detailed information concerning risks and benefits needed for this has not yet been provided to dentists by either NICE or SDCEP.

Prior to the NICE guidelines, the medico-legal position was clear. A dentist could be considered negligent if they failed to prescribe AP before IDPs for a patient at increased IE risk, if that patient went on to develop IE. After the 2008 NICE guidelines, dentists could be considered negligent if they prescribed AP against NICE advice and the patient suffered an adverse drug reaction (ADR). Indeed, dentists were informed they would be in breach of their NHS contracts if they did not follow NICE guidance and dental defence organisations threatened to withdraw cover for adverse events following the use of AP.

## Impact of the change in the law on consent

Following changes to the law on consent,^22^^,^^23^ it has become essential that dentists inform all patients at increased risk of IE (both moderate- and high-risk) of the risk posed by IDPs before any dental treatment, and of the potential risks and benefits of AP so that patients can make their own treatment choice. Not to do so would leave the dentist liable to legal challenge if the patient developed either an ADR or IE. Dentists have therefore been placed in a difficult position by the lack of clear NICE guidance and the paucity of information from either NICE or SDCEP concerning the risk of developing IE following IDPs or the risks/benefits of AP. Without this information, how can they properly inform their discussions with patients? This leaves dentists in an invidious position. Meanwhile, patients are expected to make difficult decisions for which they are ill-informed and ill-equipped.

## New evidence

### AHA and ESC guideline reviews

Taking account of NICE's position, the AHA again reviewed their guidance in 2021 but found no reason to change their advice that AP should be used for high-risk patients undergoing IDPs.^24^ The ESC has also undertaken an extensive review of the evidence and published updated guidelines in August 2023.^25^ They strengthened their recommendation that those at high risk should receive AP before IDPs as a result of the extent and quality of new evidence that has become available since their last review in 2015 (from Class IIa [weight of evidence/opinion is in favour of usefulness/efficacy] to Class I [evidence and/or general agreement that a given treatment or procedure is beneficial, useful, effective]). Patients with ventricular assist devices were added to the list of those at high risk, with the recommendation that they should receive AP before IDPs (Class IIa), along with heart transplant recipients (Class IIb). Although the ESC guidelines continue to say that AP is not routinely recommended for those at moderate/intermediate IE risk, they do now suggest it may be considered on an individual basis.^25^

Fifteen years after its guidance against the use of AP to prevent IE, NICE therefore remains isolated in the view that high-risk UK patients should not receive the same AP protection from IE that is provided elsewhere in the world.

### Changes in NICE guideline methodology

NICE has changed its guideline development methodology in two important respects to: i) accept that a rigid reliance on RCT evidence may be inappropriate when that evidence is unavailable or unrealistic (as is the case with AP prevention of IE);^26^^,^^27^ and ii) acknowledge that decisions cannot be based on cost-effectiveness alone.^26^^,^^27^ Given these changes, it is time for NICE to review its guidance.

## New research evidence

Since 2015, much new research evidence has emerged in the following areas: the risk of ADRs with AP; the cost-effectiveness of AP; the link between IDPs and IE; AP efficacy; and the importance of good oral hygiene.

### Risk of adverse reactions

The two main factors that caused NICE to recommend against AP were the lack of evidence of efficacy and concerns regarding the risk of ADR. However, their measures of the risk of an ADR were substantially overestimated (20 fatal ADR/million prescriptions^15^^,^^28^[relying on data from 1968 and 1984]^29^^,^^30^ and 20,000 non-fatal ADR/million prescriptions [derived from a 1997 estimate]).^28^^,^^31^ Furthermore, these were estimates of the ADR risk following any dose, duration, or route of administration (intravenous, intramuscular, oral), of any type of penicillin used for any purpose (including treating existing infections), rather than the risk associated with a single oral dose of amoxicillin. Nevertheless, based on these historical data, NICE concluded that ‘AP against IE for dental procedures may lead to a greater number of deaths through fatal anaphylaxis than a strategy of no AP, and is not cost-effective'.^15^

In 2015, soon after the NICE guideline review, new UK-based evidence demonstrated that the ADR risk following a single 3 g oral dose of amoxicillin used for AP was substantially lower than NICE estimates. In one study, no fatal ADR were identified following three million amoxicillin AP prescriptions and only 22.6 non-fatal ADR/million prescriptions,^32^ while a second study demonstrated no ADR deaths following use of a single 3 g oral dose of amoxicillin for AP.^33^ The NICE ADR estimates therefore significantly overestimated the ADR risk posed by amoxicillin AP used for IE prevention.

### Cost-effectiveness

Lack of evidence for AP efficacy and over-estimation of ADR risk led NICE to calculate that use of AP to prevent IE was not cost-effective. In 2016, however, a new health economic analysis using AP-specific ADR data found that AP only had to prevent 1.4 IE cases a year in those at high risk to be cost-effective.^34^ The same analysis also suggested that the NHS in England would save £5.5-8.2 million and achieve health gains of >2,600 quality adjusted life-years annually if AP were re-instated for those at high risk.

### The link between IDPs and IE

Since the 2015 NICE guideline review, several studies have investigated the association between IDPs and IE. Most have been too small to detect any association or were performed in countries where any association could be hidden, since high-risk patients were recommended to receive AP. Despite these limitations, a study from Korea (where AP is recommended in high-risk patients) found that IDPs in patients with implanted cardiac electrical devices were associated with a significantly increased risk of IE (OR: 1.75; 95% CI: 1.48-2.05; p <0.001).^35^ Similarly, a self-controlled case series study from Taiwan (where AP is also recommended) also identified a significant association between IDPs and IE (age-adjusted incidence rate-ratio: 1.14; 95% CI: 1.02-1.26).^36^

A study from France (where AP is recommended for those at high risk) comparing IDP incidence in the three months before the development of IE in 73 patients with OVGS-IE and 192 controls with IE caused by other bacteria found that OVGS-IE patients were significantly more likely to have undergone IDPs in the preceding three months (OR: 3.31; 95% CI: 1.18-9.29).^37^ Another French study of 648 patients with prosthetic heart valves who developed OVGS-IE found a significant association between IDPs and the development of IE in the three months following the dental procedure (OR: 1.66; 95% CI: 1.05-2.63; p = 0.03).^38^

Despite being performed in countries where AP is recommended, these studies suggest a significant association between IDPs and subsequent IE. Since AP is not recommended in the UK, any association between IDPs and IE should be maximally exposed. A study using NHS general dental practice records was therefore attempted but database limitations made the study impossible.^39^ However, the same problem did not apply to IDP recording in the hospital outpatient setting, where a significant association was found between dental extractions and surgical tooth removal and the subsequent development of IE (OR: 2.14; 95% CI: 1.22-3.76; p <0.05).^40^

Although none of these studies were able to distinguish whether a specific IDP was covered by AP (or not), two recent US-based studies were able to address this deficit.^41^^,^^42^ The first included patients with employer-provided medical, dental and prescription benefits cover (essentially employer-provided private medical and dental insurance) and performed both case-crossover (eliminating selection bias and confounding) and cohort analyses.^41^ In the case-crossover analysis of 3,774 patients who developed IE, there was a significant association between the development of IE and IDPs undertaken in the preceding four weeks for high-risk patients (OR: 2.00; 95% CI: 1.59-2.52; p = 0.002). This association was particularly strong for dental extractions (OR: 11.08; 95% CI: 7.34-16.74; p <0.0001) and oral surgery procedures (OR: 50.77; 95% CI: 20.79-123.98; p <0.0001). The cohort analysis of almost eight million patients also found that the odds of developing IE were significantly increased following extractions (OR: 9.22; 95% CI: 5.54-15.88; p <0.0001) and oral surgical procedures (OR: 20.18; 95% CI: 11.22-36.74) in high-risk individuals.^41^

A very similar study was performed in US Medicaid patients with the most basic medical and dental cover.^42^ Case-crossover analysis of 2,647 IE cases confirmed the association between IDPs and the subsequent development of IE for those at high risk, particularly after extractions (OR: 3.74; 95% CI: 2.65-5.27; p <0.005) and oral surgical procedures (OR: 10.66; 95% CI: 5.18-21.92; p <0.0001). The 1.68 million patient cohort study also found an increased IE incidence after IDPs in high-risk patients, particularly after extractions (OR: 14.17; 95% CI: 5.40-52.11; p <0.0001) and oral surgical procedures (OR: 29.98; 95% CI: 9.62-119.34).^42^ High-risk Medicaid patients had a six times higher incidence of IE following IDPs than those with employer-provided private medical/dental insurance cover, presumably as a result of differences in general and dental health, access to medical/dental care and compliance with AP guidelines in these two populations.^42^

Together, these studies provide strong new evidence for an association between IDPs and the development of IE.

### AP efficacy

**Fig. 2:**
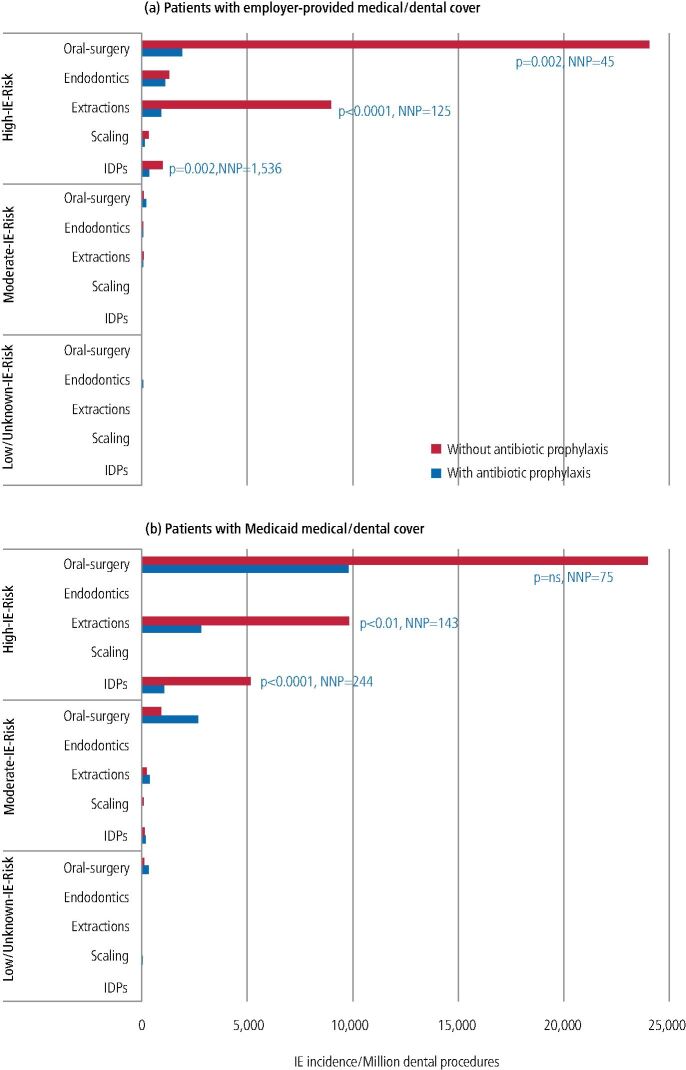
IE incidence in individuals at high, moderate or low risk of IE following IDPs, or IDPs of different types, performed with or without AP cover. Study data from two different populations: a) patients with employer-provided medical/dental cover (reprinted from *Journal of the American College of Cardiology*, Vol 80, Thornhill *et al*., ‘Antibiotic Prophylaxis Against Infective Endocarditis Before Invasive Dental Procedures' pp 1029-1041, 2022, with permission from Elsevier);^41^ and b) those with Medicaid medical/dental cover (reprinted from Thornhill *et al*., ‘Endocarditis, invasive dental procedures, and antibiotic prophylaxis efficacy in US Medicaid patients', *Oral Diseases*, 2023, Wiley).^42^ P-values compare IE incidence with and without AP cover (p = ns where no p-value shown). NNP = number needed to prevent (that is, the number of dental procedures that need AP cover to prevent one IE case). IE risk status based on ESC and AHA guidelines (see Box 1)

Since these studies could distinguish whether specific dental procedures were covered by AP (or not), they were also able to investigate whether AP could significantly reduce the incidence of IE following IDPs. In patients with employer-provided medical/dental cover, AP significantly reduced IE incidence following IDPs (OR: 0.38; 95% CI: 0.22-0.62; p = 0.002), and particularly extractions (OR: 0.13; 95% CI: 0.03-0.34; p <0.0001) or oral surgical procedures (OR: 0.09; 95% CI: 0.01-0.35; p = 0.002) in those at high risk (but not moderate or low risk [Figure 2a]).^41^ AP also significantly reduced the incidence of IE following IDPs in high-IE-risk Medicaid patients (OR: 0.20; 95% CI: 0.06-0.53; p <0.0001), particularly following extractions (OR: 0.29; 95% CI: 0.08-0.77; p <0.01 [Figure 2b]).^42^ These studies calculated that the number of IDPs, extractions or oral surgical procedures that needed AP cover to prevent one case of IE (the number needed to prevent [NNP]) was 1,536, 125 and 45, respectively, for those with employer-provided medical/dental cover and 244, 143 and 71 for Medicaid patients (Fig. 2).^42^ In other words, one IE case would be prevented for every 45 oral surgical procedures performed under AP cover in high-risk patients with employer-provided medical/dental insurance cover.

For the first time, these studies provide powerful evidence of AP efficacy in reducing the risk of IE in high-risk patients undergoing IDPs, thereby supporting ESC and AHA guideline recommendations that high-risk patients should receive AP before undergoing IDPs. While not RCTs, these large observational studies come close and provide new evidence that should be considered by NICE. They also provide hard data on the risk of IE following IDPs and the risks and benefits of AP that dentists can use to inform their discussions with patients as advised by SDCEP^21^ and NICE.^18^ (See accompanying article: ‘Prevention of infective endocarditis in at risk patients:how should dentists proceed in 2024?' for further practical advice).^7^

### The importance of good oral hygiene

Over the last 20 years, the debate over the cause of oral bacteria-related IE has divided into two camps: those who attribute cases to the bacteraemia caused by daily oral activities, for example, toothbrushing, flossing and mastication, and those who attribute them to bacteraemia caused by IDPs (the former view contributing to the NICE decision to recommend against AP in 2008). Currently, however, there are no data comparing the risk of developing IE from daily activities and IDPs. A recent systematic review showed that bacteria can enter the circulation in both scenarios, although bacteraemia was more likely following dental extractions (62-66% frequency) and other IDPs, and lower following toothbrushing (8-26% frequency), flossing and chewing (16% frequency).^43^ Bacterial load and duration of bacteraemia are also likely to be important in determining the risk of IE and most studies have found a longer duration of bacteraemia following IDPs than during daily oral activities.^43^ While IDPs are likely to result in high intensity intermittent bacteraemia and daily oral activities are likely to result in frequently repeated lower intensity exposure, no studies have addressed which is more likely to result in IE. It is important, therefore, to acknowledge that both mechanisms have potential to cause IE and to focus prevention strategies accordingly.

While AP seems to be effective in reducing IE following IDPs in high-risk patients, it would clearly be impractical for preventing the threat posed by daily activities. Furthermore, IE related to both IDPs and daily activities is likely to be greater in those with poor oral hygiene.^44^ Maintenance of good oral hygiene is, therefore, paramount in reducing the risk of IE and a recent clinical trial demonstrated that moderate-risk patients with markers of poor oral hygiene were significantly more likely to develop IE.^45^ The authors concluded that ‘those at risk for IE can reduce potential sources of IE-related bacteraemia by maintaining optimal oral health through regular professional dental care and oral hygiene procedures'. Of note, the advantages of good oral hygiene are important not just for those at high risk (where the benefits of AP appear to be focused) but also for those at moderate risk (who may not benefit from AP). The benefits of improved oral hygiene may also explain why scaling does not seem to pose the same level of risk as extractions and oral surgery procedures (despite being a relatively invasive procedure).

Although NICE guidelines mention the importance of maintaining oral health, the importance of good oral hygiene should be emphasised for all those at increased IE risk (moderate and high risk) at the same time as providing AP cover for those at high risk undergoing IDPs.

## Extent of the problem

In the UK, there are approximately 397,000 high-risk individuals (0.6% of the 67.3 million population) who undergo 131,033 dental procedures each year (approximately 0.33 dental procedures per person).^34^^,^^46^ Of these, 63,551 (48.5%) are IDPs,^39^ including 48,362 (76.1%) scaling procedures, 9,978 (15.7%) extractions, 1,398 (2.2%) endodontic treatments and 3,813 (6%) surgical or mixed procedures.^39^ The studies described above suggest that re-introduction of AP for those at high risk could significantly reduce the incidence of IE following IDPs. Specifically, the NNP data indicate that ~41-261 IE cases could be prevented each year in the UK (including 12-78 deaths). Given that the health economic analysis published in 2016 calculated that AP would be cost-effective if it prevented 1.4 high-risk cases each year, re-introduction of AP for those at high risk would not only save lives and improve the quality of life for many, but it would also be highly cost-effective and result in substantial NHS cost savings. Improving oral hygiene for moderate- and high-risk patients would reduce the incidence of IE further still and produce further savings.

## Conclusions and call for action

All studies described herein have been published since the last review of NICE guidance in 2015 and demonstrate the importance of improving oral hygiene, confirm the association between IDPs and IE, and prove that AP is safe, efficacious and cost-effective. They provide important new evidence that AP reduces the risk of IE following IDPs in high-risk patients and strongly support the ESC and AHA guidelines.

In the light of this new evidence, the potential to save lives, improve patient care and save scarce NHS resources, we believe that a review of NICE guidance is now essential to allow a consistent international approach to the prevention of IE and its complications.

Finally, as part of any review it is essential NICE looks closely at including detailed information for dentists to use when having informed consent discussions with patients, and that guidance is unambiguous and clinically useful.
